# Pollen Load and Flower Constancy of Three Species of Stingless Bees (Hymenoptera, Apidae, Meliponinae)

**DOI:** 10.21315/tlsr2017.28.2.13

**Published:** 2017-07-31

**Authors:** Norita Widya Pangestika, Tri Atmowidi, Sih Kahono

**Affiliations:** 1 Department of Biology, Faculty of Mathematics and Natural Sciences, Bogor Agricultural University, Dramaga Campus, Bogor 16680, Indonesia; 2 Zoology Division, Research Center for Biology-LIPI, Bogor 16911, Indonesia

**Keywords:** Bees, Stingless Bees, Flower Constancy, Pollen, Pollen Load

## Abstract

The genera of stingless bees play an important role as pollinators of plants. These bees are actively involved in the pollination of agricultural crops and known to have preferences in selecting flowers to pollinate. The aims of this study were to analyse the pollen load and flower constancy in *Tetragonula laeviceps*, *Lepidotrigona terminata*, and *Heterotrigona itama*. Each individual of species stingless bees collected and was put in a 1.5 mL micro-tube contain 0.5 mL 70% ethanol:glycerol (4:1). Pollen loads on each individual of stingless bees was counted by hemocytometer. Flower constancy of stingless bees was measured based on percentage of pollen type loaded on the body. Results showed that the pollen loads of *H. itama* was the highest (31392 pollen grains) followed by *L. terminata* (23017 pollen grains) and *T. laeviceps* (8015 pollen grains). These species also demonstrated different flower constancy, *T. laeviceps* on Poaceae flowers (76.49%), *L. terminata* on Euphorbiaceae flowers (80.46%), and *H. itama* on Solanaceae flowers (83.33%).

## INTRODUCTION

Although many flowering plants are capable to self-pollinate, cross-pollination is needed to increase genetic diversity of plants. In cross-pollination, pollens are transferred by wind, water, birds, bats, or bees. These latter are identified as the most common agents involved in cross-pollination (Davies 1988).

Pollen and nectar are attractive main sources for bees. Pollen grains have thin walls composed of exine and intine (Lersten 2004). Pollen contains protein and amino acid as nutrient sources (Szczesna 2006). Pollen size is an important parameter for pollen identification. The pollen size can be classified into very small (<10 μm), small (10–25 μm), medium (25–50 μm), large (50–100 μm), very large (100–200 μm), and gigantic (> 200 μm) (Erdtman 1972). Generally, pollen diameter is 15–60 μm (Kearns & Inouye 1993). Pollen is identified based on five principal properties: polarity, symmetry, aperture, shape and size (Erdtman 1972).

Stingless bees (Hymenoptera; Apidae; Meliponinae) are social insects (Wille 1983) that play an important role as pollinator of plants (Inoue *et al.* 1985). Bees are known to actively visit in agricultural crops, such as peppers (Cruz *et al.* 2005) and tomatoes (Santos *et al.* 2009). There are about 100 species of plants visited by 37 species of stingless bees belonging to the families of Acantaceae, Begoniaceae, Caesalpiniaceae, Cucurbitaceae, Ciperaceae, Poaceae, Malvaceae, Myrtaceae, Rutaceae, Euphorbiaceae, Leguminosae, Rubiaceae, Sapindaceae, and Solanaceae as food sources for these bees (Engel & Bakels 1980; Ramalho *et al.* (1994); Slaa *et al.* (2006)).

Stingless bee has caste system in the colony: reproductive caste (queens and males) and non-reproductive caste (workers) (Michener 2000). The bees are distributed in the tropics and subtropics areas (Free 1993) and about 50 species were found in Southeast Asia (Inoue *et al.* 1985). In Indonesia, stingless bees are also widely distributed in Kalimantan (about 31 species), Sumatra (41 species), and Java (9 species) (Schwarz 1937). Sakagami *et al.* (1990) reported six species of stingless bees in Java, i.e. *T. laeviceps*, *H. itama*, *Tetragonula drescheri*, *Tetrigona apicalis*, *Geniotrigona thoracica*, and *L. terminata.* In India, these five spesies of stingless bee were found, those are *T. bengalensis, Tetragonula iridipennis, Tetragonula ruficornis, Tetragonula laeviceps, Tetragonula praeterita* and *L. terminata* (Rahman *et al.* 2013). In Peninsular Malaysia, Salim *et al.* (2013) were found 17 species of nine genera of stingless bees, those are *Geniotrigona, Heterotrigona, Homotrigona, Lepidotrigona, Lophotrigona, Sundatrigona, Tetrigonilla, Tetragonula,* and *Tetrigona*.

These bees have spesific structure to collect pollens, including a pollen basket or corbicula located at the tibia of hind legs. The number of pollens attached on the bee body is called pollen loads. These bees also have a length variation of proboscis that is used to collect pollens and nectar when foraging on the flowers (Davies 1988). During foraging, bees also showed a flower constancy. This latter shows the preference of bees to visit flowers of a particular plant species in one trip (Waser 1986). Ramalho *et al.* (1994) reported that in one foraging, commonly stingless bees visits only one flower type on average 97% and 3% taken from other plants.

In this research, we measured pollen loads and flower constancy of three species of stingless bees, i.e. *H. itama*, *L. terminata* and *T. laeviceps*. *H. itama* has larger body size (3.0–7.5 mm), dominantly blackish body, with one weak tooth on the mandible. *L. terminata* has medium to large body size (4.0–5.5 mm), yellow-brownish body on mesoscutellum, While, *T. laeviceps* is a small species (about 3.5 mm body lenght), brown-blackish body with transparent wings (Sakagami *et al.* 1990; Michener 2007).

## MATERIALS AND METHODS

### Sample Collection

Collection of stingless bee individuals was carried out from October to November 2015 in the colonies located at Ecology Park (Cibinong Science Center-Botanical Garden) area of Indonesian Institute of Sciences (LIPI), Cibinong, Bogor, West Java, Indonesia. The species used in this study were *H. itama*, *L. terminata,* and *T. laeviceps.* Six individuals of workers that carried pollens of each species were collected to measure pollen loads and body weight.

### Pollen Removal from Stingless Bee Individuals

Each individual of worker stingless bees collected was anesthetised using ethyl acetate and then was put in a 1.5 mL micro-tube contain 0.5 mL 70% ethanol:glycerol (4:1). Then, the samples were rotated during 24 h, after which the bee was removed from the micro-tube. Afterwards, the solution was centrifuged at 2000 rpm for 10 min. The supernatant was discarded and the 0.1 mL sediment contain pollens is remained (Dafni 1992).

### Measurements of Pollens Loads on Stingless Bees

The 0.1 mL solution contain pollens is used to measure pollen load. The number of pollens in the solution were counted using a compound microscope. The same method was replicated 4–5 times. Total pollen loads in 0.1 mL was counted.

### Pollen identification and Determination of Flower Constancy

Pollen loads on body were identified based on the methods developed by Huang (1972) and Erdtman (1972). Several morphological structures were used to identify the pollens, i.e. aperture, surface pattern, polar and equatorial forms (Erdtman 1972). Measurement of polar and equatorial pollen were conducted by using a light microscope equipped with Optilab camera. Image Raster software was used to measure pollen size. The flower constancy of each bee species of stingless bee was determined based on the percentage of conspecific pollen types (Dafni 1992)

### Data Analysis

Pollens loads of each stingless bees species were shown in bar chart. Pollens loads were correlated with body weight of each stingless bees species using scatter plot. Regression analysis was used to examine the relationship between the weight of each individual of stingless bees and the number of pollens carried by using Minitab version 16 software with a 95% confidence level (α = 5%).

## RESULTS

### Body Weight of Stingless Bees

**Figure 1:**
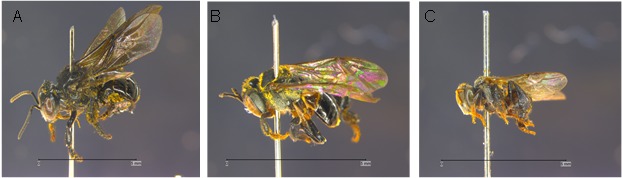
Morphological features of *H. itama* (A), *L. terminata* (B), and *T. laeviceps (*C) (scale = 5 mm).

The morphological features of three stingless bees species used in the study, i.e. *H. itama* (Fig. 1A), *L. terminata* (Fig. 1B), and *T. laeviceps* (Fig. 1C) were shown in Figure 1. There were several differences on the morphology and size of these species. The average of fresh body weight of *T. laeviceps*, *L. terminata*, and *H. itama* were 2.67 mg ± 1.01 (n = 6), 5.00 mg ± 0.53 (n = 6), and 10.55 mg ± 5.5 (n = 4), respectively.

### Pollen Loads on Stingless Bees

**Figure 2:**
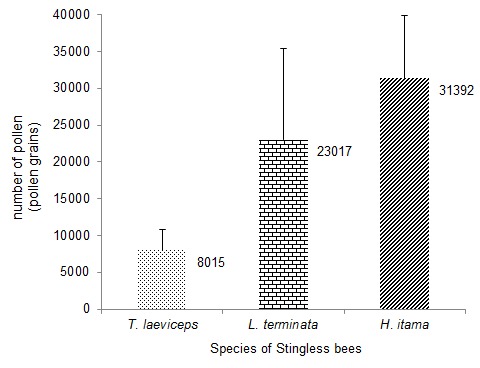
The average number of pollen load of each species of stingless bees.

**Figure 3:**
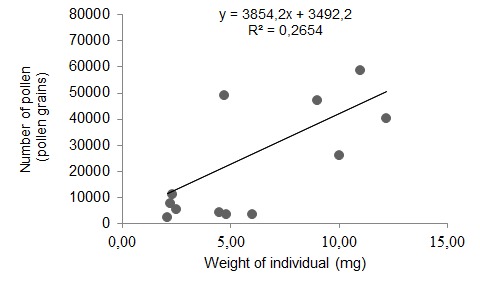
The relationship between body weight of stingless bees and the number of pollen attached on the body.

Results showed that average pollen load on each species stingless bees varied. The highest pollen loads were observed in *H. itama* (31392 pollen grains), followed by *L. terminata* (23017 pollen grains), and *T. laeviceps* (8015 pollen grains) (Fig. 2). The number of pollens attached to the body of stingless bees was positively correlated with body weight (y = 3854.2x + 3492.2, R^2^ = 0.2654, *p* = 0.017) (Fig. 3).

### The Pollen Types Attached on Stingless Bees

**Figure 4:**
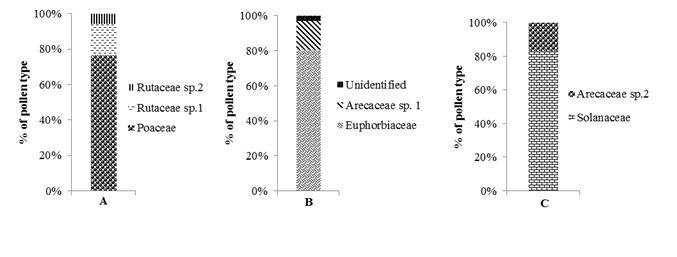
Percentage of pollen type on the body of *T. laeviceps* (A), *L. terminata* (B), and *H. itama* (C).

Pollens attached on the body of *T. laeviceps* belong to two plant families, i.e., Poaceae and Rutaceae. Dominant pollen type attached on the body of *T*. *laeviceps* was Poaceae (76.49%). The other pollen types were Rutaceae sp1 (17.89%) and Rutaceae sp2 (5.62%) (Fig. 4A). Our results showed the flower constancy of *T. laeviceps* on Poaceae flowers.

On the body of *L. terminata,* we identified three pollen types, i.e., the pollens of Euphorbiaceae, Arecaceae sp1, and unidentified pollen type. The dominant pollen type attached on the body of *L*. *terminata* was mainly Euphorbiaceae (80.46%), Arecaceae sp1 (16.67%), followed by unidentified sp1 (1.51%), unidentified sp2 (1.37%). (Fig. 4B). In *H. itama,* the dominant pollen load*s* were Solanaceae (83.33%), followed by Arecaceae sp2 (16.67%) (Fig. 4C).

## DISCUSSION

*Heterotrigona itama* has the highest body weight compared the other species used in this study. There was correlation between body size (fresh weight) and pollen loads (Veiga *et al.* 2013). *H. itama*, the biggest species used in this study, had the highest pollen loads (31392 pollen grains), whereas *T. laeviceps*, the smallest species used in this study, had the lowest pollen loads (8015 pollen grains). This result supported the study previously performed by Reinaldo (2015) that the pollen loads on *T. laeviceps* were lower than in *Apis cerana* (4228 pollen grains). The ability of the worker bees to carry the pollens also depends on the size of the pollen basket (Shuel 1992) and body size (Sadeh 2007). Pollen contains protein that is used by young colony members (Jalil & Shuib 2014).

Flower constancy is an adaptive behaviour and the best strategy for bees to reduce the energy that would be redundantly spent for choosing other plants (Gruter & Ratnieks 2011). Several factors influence flower constancy, i.e. odor or chemical compound, colors, and shapes of flowers (Gruter & Ratnieks 2011).

*Tetragonula laeviceps* showed flower constancy on the flowers of Poaceae family (76.49%). Poaceae family belongs to grass that are generally found near stingless bees colonies. In bees, foraging in a limited space increase the efficiency of foraging (Kakutani *et al.* 1993). Other individuals of stingless bees also forage on Rutaceae sp1 and Rutaceae sp2 flowers as nutrient sources. Engel and Bakels (1980) also reported that stingless bees also forage on the flowers of Caesalpiniaceae, Begoniaceae, Myrtaceae, Malvaceae and Rutaceae families.

*Lepidotrigona terminata* showed flower contancy on the flowers of Euphorbiaceae (80.46%). This result supported the study of Ramalho *et al. (*1994) demonstrating that 62%-89% of stingless bees took pollens from the Euphorbiaceae plants. Only one individual of *L. terminata* carried pollens from Arecaceae sp1 (16.67%) and the remaining individuals carried pollens from unidentified sp1 and unidentified sp2 (2.87%). Both of pollen types was unable to be identified due to the fact that the polar and equatorial forms of these pollen were not clearly visible. Polar and equatorial forms are indispensable characteristics for pollen identification (Huang 1972).

In *H. itama,* pollen loads were dominated by the pollens of Solanaceae (83.33%) and 16.67% belonged to the pollens of Arecaceae sp2 (16.67%). Some species of stingless bees were known as effective pollinators in Solanaceae (Heard 1999). In Brazil, stingless bees are effective pollinators of *Capsicum annuum* and have been reported to improve the quality and quantity of fruits (Cruz *et al.* 2005).In this study, three species of stingless bees showed different preference to visit the flowers. *T. laeviceps* prefered Poaceae plants, *L. terminata* prefered Euphorbiaceae plants, and *H. itama* prefered Solanaceae plants as their nutrient sources. It shows that the effectiveness of the pollination in some species of bees is highly dependent on the plant species thereby highlighting the importance of flower constancy in such a pollination (Kearns & Inouye 1993). Stingless bees can be used as alternatives for commercial pollination (Heard 1999), because they do not die after reproducing, have no sting, and are more resistent to pests and parasites than honey bees. Stingless bees can be used to pollinate the plants cultivated in residential areas and in the greenhouses. These bees have also been shown to successfully pollinated some greenhouse plants in temperate areas, such as Netherlands and Japan. In general, stingless bees have been demonstrated to be good alternatives as pollinators for cultivation plants (Slaa *et al.* 2006).

## CONCLUSION

Pollen loads on three species of stingless bees were varied. Pollen load on *H. itama* was highest (31392 pollen grains), followed by *L. terminata* (23017 pollen grains) and *T. laeviceps* (8015 pollen grains). The number of pollens attached to the body of stingless bees was positively correlated with body weight. Three species of stingless bees also showed different flower constancy. *T. laeviceps* showed flower contancy on the flowers of Poaceae plant (76.49%), *L. terminata* on the flowers of Euphorbiaceae (80.46%), and *H. itama* on the flowers of Solanaceae (83.33%).

## Figures and Tables

**Figure 5:**
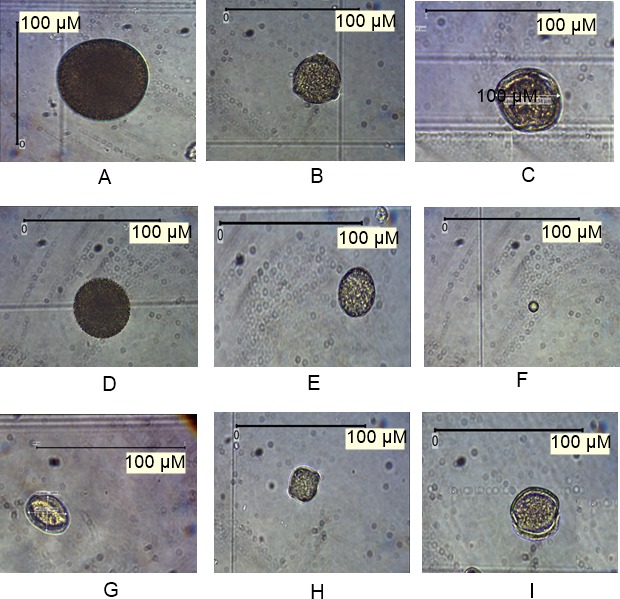
Pollen type attached on stingless bees: Pollen of Poaceae (A), Rutaceae sp1 (B), Rutaceae sp2 (C), Euphorbiaceae (D), Unidentified sp1 (E), Unidentified sp2 (F), Arecaceae sp1 (G), Solanaceae (H), Arecaceae sp2 (I).
